# Comparing Anesthesia and Surgery Controlled Time for Primary Total Knee and Hip Arthroplasty Between an Academic Medical Center and a Community Hospital: Retrospective Cohort Study

**DOI:** 10.2196/45126

**Published:** 2024-02-26

**Authors:** Thy B Nguyen, Nathaen Weitzel, Craig Hogan, Rachel M Kacmar, Kayla M Williamson, Jack Pattee, Vesna Jevtovic-Todorovic, Colby G Simmons, Adeel Ahmad Faruki

**Affiliations:** 1 University of Colorado School of Medicine Aurora, CO United States; 2 Department of Anesthesiology University of Colorado Anschutz Medical Campus Aurora, CO United States; 3 Department of Orthopaedic Surgery University of Colorado Anschutz Medical Campus Aurora, CO United States; 4 Department of Biostatistics and Informatics Colorado School of Public Health University of Colorado - Anschutz Medical Campus Aurora, CO United States; 5 Department of Anesthesiology and Perioperative Medicine MD Anderson Cancer Center Houston, TX United States

**Keywords:** anesthesia controlled time, surgery-controlled time, total joint arthroplasty, healthcare operations, efficiency, total joint replacement, knee, hip, arthroplasty, anesthesia, surgery, surgical duration, community hospital, surgeon, reliability, operating room, anesthesiology, orthopedics, perioperative, medicine

## Abstract

**Background:**

Osteoarthritis is a significant cause of disability, resulting in increased joint replacement surgeries and health care costs. Establishing benchmarks that more accurately predict surgical duration could help to decrease costs, maximize efficiency, and improve patient experience. We compared the anesthesia-controlled time (ACT) and surgery-controlled time (SCT) of primary total knee (TKA) and total hip arthroplasties (THA) between an academic medical center (AMC) and a community hospital (CH) for 2 orthopedic surgeons.

**Objective:**

This study aims to validate and compare benchmarking times for ACT and SCT in a single patient population at both an AMC and a CH.

**Methods:**

This retrospective 2-center observational cohort study was conducted at the University of Colorado Hospital (AMC) and UCHealth Broomfield Hospital (CH). Cases with current procedural terminology codes for THA and TKA between January 1, 2019, and December 31, 2020, were assessed. Cases with missing data were excluded. The primary outcomes were ACT and SCT. Primary outcomes were tested for association with covariates of interest. The primary covariate of interest was the location of the procedure (CH vs AMC); secondary covariates of interest included the American Society of Anesthesiologists (ASA) classification and anesthetic type. Linear regression models were used to assess the relationships.

**Results:**

Two surgeons performed 1256 cases at the AMC and CH. A total of 10 THA cases and 12 TKA cases were excluded due to missing data. After controlling for surgeon, the ACT was greater at the AMC for THA by 3.77 minutes and for TKA by 3.58 minutes (*P*<.001). SCT was greater at the AMC for THA by 11.14 minutes and for TKA by 14.04 minutes (*P*<.001). ASA III/IV classification increased ACT for THA by 3.76 minutes (*P*<.001) and increased SCT for THA by 6.33 minutes after controlling for surgeon and location (*P*=.008). General anesthesia use was higher at the AMC for both THA (29.2% vs 7.3%) and TKA (23.8% vs 4.2%). No statistically significant association was observed between either ACT or SCT and anesthetic type (neuraxial or general) after adjusting for surgeon and location (all *P*>.05).

**Conclusions:**

We observed lower ACT and SCT at the CH for both TKA and THA after controlling for the surgeon of record and ASA classification. These findings underscore the efficiency advantages of performing primary joint replacements at the CH, showcasing an average reduction of 16 minutes in SCT and 4 minutes in ACT per case. Overall, establishing more accurate benchmarks to improve the prediction of surgical duration for THA and TKA in different perioperative environments can increase the reliability of surgical duration predictions and optimize scheduling. Future studies with study populations at multiple community hospitals and academic medical centers are needed before extrapolating these findings.

## Introduction

Hip and knee osteoarthritis (OA) are pervasive causes of disability and pain globally, and the burden of OA is expected to increase due to population aging and the rising prevalence of obesity [1]. Total knee arthroplasty (TKA) and total hip arthroplasty (THA) are 2 of the most common and well-accepted surgical interventions to improve quality of life for patients with end-stage joint deterioration [2]. Therefore, a considerable increase has been projected for TKA and THA cases (673% and 174%, respectively) from 2005 to 2030 in the United States [3]. The anticipated demand for joint replacements combined with the importance of the operating room (OR) in hospital revenue and margins emphasize the importance of identifying factors that decrease cost and maximize efficiency in the OR [4,5]. One such process is establishing benchmarks that are accurate predictors of surgical duration in order to improve hospital operations, optimize OR schedule modeling and management, reduce health care costs, and improve patient satisfaction and experience.

Prior efforts have been made to assess OR efficiency using mean anesthesia-controlled time (ACT) and surgery-controlled time (SCT) values [6]. ACT is defined as the sum of the time starting when the patient enters the OR until the patient is ready for surgical positioning, added to the time starting when the incision is closed and ending when the patient leaves the OR [7]. SCT is defined as the time from when the patient is ready for positioning to when the surgical sites are closed. Studies examining SCT for TKA found that computer-based estimations of historical performance were a better predictor of actual SCT than the estimates provided by surgeons, while assessments of heterogeneity of ACT and SCT based on current procedural terminology (CPT) codes have also highlighted the need for more granular prediction models [8,9]. Moreover, ACT and SCT at academic institutions may be increased because of teaching responsibilities for anesthesia and surgery trainees and may not reflect mean ACT and SCT for the same procedures in other settings. Furthermore, a spectrum of clinical and nonclinical factors could contribute to significant variation in case duration between surgeons [10,11]. This study will compare the ACT and SCT of THA and TKA between an academic medical center (AMC) and a community hospital (CH) for 2 orthopedic surgeons.

We hypothesize that after adjusting for surgeon, the ACT and SCT between an AMC and a CH will have a statistically significant difference for both knee and hip procedures.

## Methods

### Design

This retrospective 2-center observational cohort study was conducted at an AMC—the University of Colorado Hospital—and a university-affiliated CH—UCHealth Broomfield Hospital. Prior to the COVID-19 pandemic, hip and knee replacement surgeries were primarily performed at the AMC. However, during the pandemic, these surgeries were relocated to the CH from March 2020 through August 2020 and again in November 2020. Both orthopedic surgeons work with the same team of orthopedic surgery physician assistants and trainees (residents and fellows) at both locations. The University of Colorado Department of Anesthesiology staffs both the AMC and CH with an anesthesia care-team model consisting of supervising attending physicians and anesthesia providers such as certified registered nurse anesthetists, anesthesiology assistants (AAs), or anesthesiology resident physicians-in-training. The academic center also has student AAs who often work alongside certified registered nurse anesthetists and AAs. The CH does not have anesthesiology residents or student AAs present for any procedure. The practice for anesthesiology at both locations includes primarily performing neuraxial anesthesia on both TKA and THA if patients are appropriate and amenable to this type of anesthetic. For TKA, single-shot adductor canal blocks were performed in the preoperative area before the patient was brought to the OR. In the OR, the neuraxial anesthetic or a general anesthetic was performed.

### Eligibility Criteria

Inclusion criteria for the study included participants undergoing primary THA and TKA. These cases were performed by 2 fellowship-trained adult reconstructive orthopedic joint surgeons who operated at both the AMC and CH. The time frame for cases performed was from January 1, 2019, to December 31, 2020. Inclusion criteria included being aged older than 18 years and the procedure type was determined based on CPT codes billed for the case. Only CPT codes 27130 (THA) and 27447 (TKA) were assessed in this study. Exclusion criteria included cases with missing data required to calculate ACT and SCT.

### Data Collection and Storage

Demographic data and time stamps for each case were collected from electronic medical records and stored securely on the AMC’s cloud drive.

### ACT and SCT Calculation

The time stamps for *In Room Time*, *Ready for Positioning and Prep Time*, *Incision Time*, *Close Time*, and *Out of Room Time* were collected for each case. *Ready for Positioning* is defined as the point when the anesthesia team has completed their activities, signifying that the patient was prepared for surgical positioning. *Ready for Positioning and Prep Time* indicated that all presurgical anesthesia-related activities were completed and the surgical team could begin positioning the patient and performing surgical preparation. ACT was calculated based on *([Ready for Positioning and Prep Time] – [In Room Time]) + ([Out of Room Time] – [Close Time])*. SCT was calculated based on *([Close Time] – [Ready for Positioning and Prep Time])*.

### Statistical Analysis

Descriptive statistics were performed using means and SDs for continuous variables, whereas counts and percentages were used for categorical variables. The primary outcome was the duration of ACT and SCT. Several independent variables were investigated for association with ACT and SCT in TKA and THA procedures. These independent variables include the location (AMC vs CH), surgeon identity (1 of 2 surgeons), American Society of Anesthesiologists (ASA) classification (dichotomized into ASA class I/II, representing mild to moderate systemic disease, vs ASA class III/IV, representing severe systemic disease), and anesthesia type (general vs neuraxial). Several multiple regressions were fit to assess relevant associations. The first tested association describes 4 multivariable linear regressions; for each outcome (ACT or SCT), separate multivariable linear regressions were fit for each surgery type (TKA or THA). Location and surgeon identity were included as independent variables. The second tested association is of 4 separate multivariable regressions; however, the set of modeled independent variables changes including location, surgeon identity, and ASA classification as covariates. The third tested association is of 4 separate multivariable regressions using location, surgeon identity, and anesthetic type as covariates.

Associations were considered statistically significant if the *P* values were less than α at the .05 level. *R*^2^ and adjusted *R*^2^ are reported for multivariable regressions. *R*^2^ characterizes the proportion of variability in the outcome explained by model covariates, thus providing an estimate of the predictive utility of the model. Adjusted *R*^2^ likewise estimates the model’s predictive usefulness, with a correction for the number of independent variables. R (version 4.0.4; R Core Team ) was used for all analyses.

### Ethical Considerations

The study was reviewed by the University of Colorado Denver Institutional Review Board and the study was approved for exempt status (Colorado Multiple Institutional Review Board Protocol 20-2987), as it involved an observational retrospective analysis of existing medical records and therefore did not require additional interventions or the collection of new data from human research participants. Given the exempt status of the study, the written consent requirements of participants were waived for this Colorado Multiple Institutional Review Board Protocol. The original informed consent for the primary data collection allowed for secondary analyses without additional consent, as approved by the institutional review board. This study was designed and executed following the STROBE (Strengthening the Reporting of Observational Studies in Epidemiology) guidelines for cohort studies (Multimedia Appendix 1). To ensure the confidentiality and privacy of human research participant data, all patient records used in this study were deidentified prior to analysis. As there were no interactions or additional interventions with the participants, compensation was not applicable, and therefore not provided.

## Results

**Figure 1:**
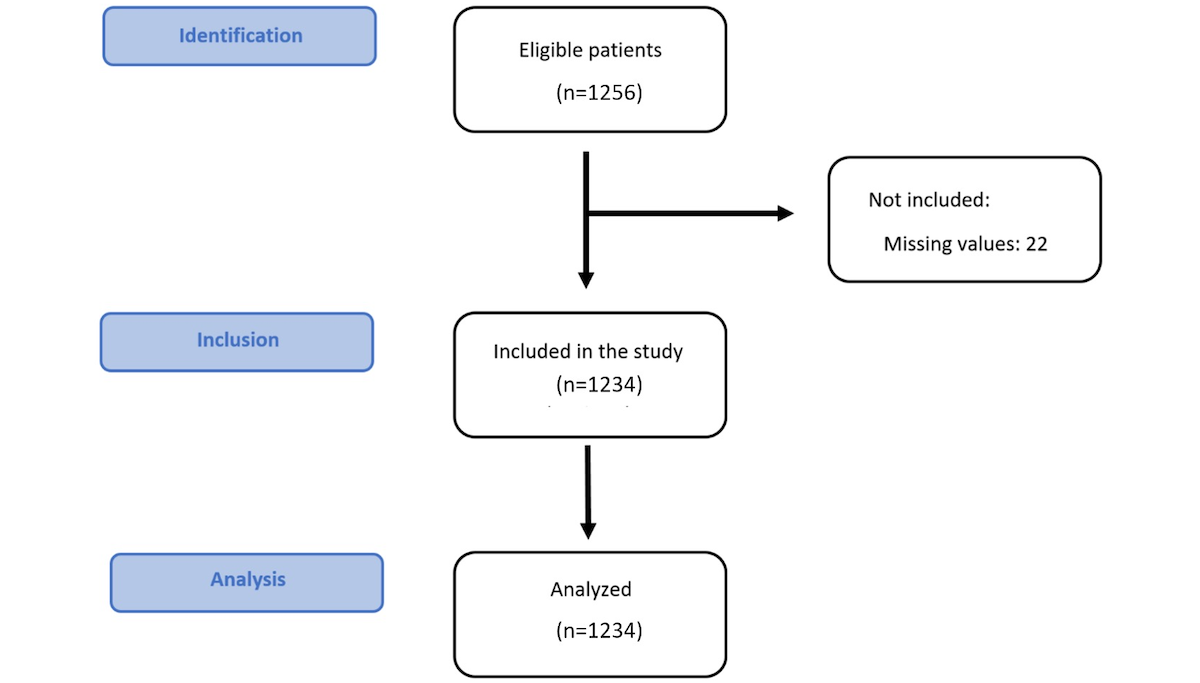
STROBE (Strengthening the Reporting of Observational Studies in Epidemiology) flow diagram.

There were 1256 observations for the 2 surgeons at the AMC and CH from January 1, 2019, to December 31, 2020. There were 619 THA observations and 637 TKA observations. A total of 10 (1.6%) out of 619 THA cases and 12 (1.8%) out of 637 TKA cases had missing values and were excluded from the analyses (Figure 1). One TKA case was missing ASA classifications and was omitted for regression controlling for this variable. The data set included 21 bilateral procedures at the AMC and 3 bilateral procedures at the CH. Secondary CPT codes were documented for a total of 5 cases including 1 cystoscopy, 1 tendon repair, 2 arteriograms, and 1 total hip liner exchange. All of the cases with secondary CPT codes documented occurred at the AMC.

There were no significant differences between the AMC and CH patient groups for age, sex, and ASA classification (all *P*>.05; Table 1). For THA, 29.2% (130/445) of the cases performed at the AMC used general anesthesia, while 7.3% (12/164) of the cases performed at the CH used general anesthesia, despite no statistically significant difference in ASA classification. Results were similar for TKA, as 23.8% (109/457) of the cases performed at the AMC used general anesthesia, while 4.2% (7/168) of the cases performed at the CH used general anesthesia, despite no statistically significant difference in ASA classification. The observed average SCT was 14.61 minutes longer for surgeon 1 and 9.31 minutes longer for surgeon 2 at the AMC in comparison to the CH for THA procedures. Furthermore, the observed average SCT was 18.01 minutes longer for surgeon 1 and 14.37 minutes longer for surgeon 2 at the AMC in comparison to the CH for TKA procedures (Table 2). The values for ACT also consistently showed increased time at the AMC for both THA and TKA cases for both surgeons (Table 2).

**Table 1:** Patient demographics and case characteristics.

Characteristics				Cases performed at AMC^a^ (n=902)		Cases performed at CH^b^ (n=332)		*P* value^c^
**Patient demographics**								
	Age (years), mean (SD)			63.1 (12.5)		63.5 (10.4)		.59
	Female sex, n (%)			n (59.1)		n (60.5)		.70
**Procedure and its ASA^d^ classification, n (%)**								
	**THA^e^ (AMC: n=445; CH: n=164)**							
		I/II	266 (59.8)		110 (67.1)		.12	
		III/IV	179 (40.2)		54 (32.9)		N/A^f^	
	**TKA^g^ (AMC: n=457; CH: n=168)**							
		I/II	261 (57.1)		107 (63.7)		.17	
		III/IV	195 (42.7)		61 (36.3)		N/A	
**Procedure and its anesthetic classification, n (%)**								
	**THA (AMC: n=445; CH: n=164)**							
		General anesthesia	130 (29.2)		12 (7.3)		<.001	
		Neuraxial anesthesia	315 (70.8)		152 (92.7)		N/A	
	**TKA (AMC: n=457; CH: n=168)**							
		General anesthesia	109 (23.8)		7 (4.2)		<.001	
		Neuraxial anesthesia	348 (76.2)		161 (95.8)		N/A	
Missing documentation			1 (0.2)		0 (0)		N/A	

^a^AMC: academic medical center.

^b^CH: community hospital.

^c^*P* values correspond to a hypothesis test for the association of the study variable with surgical location. Continuous variables are assessed via 2-tailed *t* test and dichotomous variables via a difference of proportions test.

^d^ASA: American Society of Anesthesiologists.

^e^THA: total hip arthroplasty.

^f^N/A: not applicable.

^g^TKA: total knee arthroplasty.

**Table 2:** Comparison of the mean (SD) ACT^a^ and SCT^b^ for total hip arthroplasty and total knee arthroplasty between surgeons and between operative settings.

Outcome and variable		Total hip arthroplasty		Total knee arthroplasty	
		AMC^c^, mean (SD)	CH^d^, mean (SD)	AMC, mean (SD)	CH, mean (SD)
**ACT (min)**					
	Surgeon 1	27.03 (12.97)	24.07 (8.01)	24.91 (11.34)	20.29 (7.72)
	Surgeon 2	25.18 (10.69)	20.98 (8.67)	22.71 (8.34)	20.51 (7.42)
**SCT (min)**					
	Surgeon 1	116.46 (27.03)	101.85 (25.08)	116.49 (25.56)	102.63(18.45)
	Surgeon 2	111.96 (31.7)	102.61 (23.03)	106.26 (43.72)	91.99 (16.28)

^a^ACT: anesthesia-controlled time.

^b^SCT: surgery-controlled time.

^c^AMC: academic medical center.

^d^CH: community hospital.

Location and surgeon identity were included as independent variables. After adjusting for surgeon, the mean ACT for THA at the AMC was 3.77 (95% CI 1.83-5.71) minutes longer than for the CH and 3.58 (95% CI 1.91-5.26) minutes longer for TKA (both *P*<.001; Table 3). After adjusting for surgeon, the mean SCT at the AMC was 11.14 (95% CI 6.02-16.26) minutes longer for THA and 14.04 (95% CI 8.43-19.65) minutes longer for TKA (both *P*<.001; Table 3) in comparison to the CH. Having a moderate to severe systemic disease (ASA class III/IV) increased the ACT by 3.76 (95% CI 2.00-5.51; *P*<.001) minutes and SCT by 6.33 (95% CI 1.66-10.99; *P*=.008) minutes for THA after adjusting for location and surgeon (Table 4). Having an ASA classification of III/IV did not significantly increase the ACT time for TKA (*P*=.08; Table 4). There was no significant difference noted for ACT and SCT between neuraxial anesthesia and general anesthesia (all *P*>.05; Table 5). For all models, the adjusted *R*^2^ was less than 10%, indicating that a significant amount of the variation in ACT and SCT is not explained by hospital, surgeon, ASA classification, or anesthetic used.

**Table 3:** Multivariable linear regression coefficients for the association of ACT^a^ and SCT^b^ with hospital and surgeon.

Outcome and variable			Total hip arthroplasty^c^							Total knee arthroplasty^d^				
			Estimates (min)		95% CI		*P* value		Estimates (min)			95% CI		*P* value
**ACT**														
	Coefficient intercept	23.47		21.42 to 25.51		<.001		21.03			19.47 to 22.58		<.001	
	AMC^e^	3.77		1.83 to 5.71		<.001		3.58			1.91 to 5.26		<.001	
	Surgeon 1	–2.18		–3.98 to –0.38		.02		–1.57			–3.06 to –0.08		.04	
**SCT**														
	Coefficient intercept	104.43		99.04 to 109.83		<.001		102.50			97.29 to 107.71		<.001	
	AMC	11.14		6.02 to 16.26		<.001		14.04			8.43 to 19.65		<.001	
	Surgeon 1	–3.12		–7.87 to 1.63		.20		–10.34			–15.33 to –5.35		<.001	

^a^ACT: anesthesia-controlled time.

^b^SCT: surgery-controlled time.

^c^ACT for total hip arthroplasty had 609 observations and an *R*^2^/*R*^2^ adjusted value of 0.033/0.030; and total knee arthroplasty had 625 observations and an *R*^2^/*R*^2^ adjusted value of 0.033/0.029.

^d^SCT for total hip arthroplasty had 609 observations and an *R*^2^/*R*^2^ adjusted value of 0.032/0.029; and total knee arthroplasty had 625 observations and an *R*^2^/*R*^2^ adjusted value of 0.058/0.055.

^e^AMC: academic medical center.

**Table 4:** Multivariable linear regression coefficients for the association of ACT^a^ and SCT^b^ with ASA^c^, hospital, and surgeon.

Outcome and variable			Total hip arthroplasty^d^							Total knee arthroplasty^e^				
			Estimates (min)		95% CI		*P* value		Estimates (min)			95% CI		*P* value
**ACT**														
	Coefficient intercept	22.13		20.02 to 24.24		<.001		20.53			18.87 to 22.18		<.001	
	ASA class III/IV	3.76		2.00 to 5.51		<.001		1.33			–0.18 to 2.84		.08	
	AMC^f^	3.50		1.58 to 5.42		<.001		3.50			1.82 to 5.18		<.001	
	Surgeon 1	–2.03		–3.81 to –0.25		.03		–1.53			–3.02 to –0.03		.045	
**SCT**														
	Coefficient intercept	102.18		96.56 to 107.80		<.001		101.56			96.02 to 107.11		<.001	
	ASA class III/IV	6.33		1.66 to 10.99		.008		2.61			–2.46 to 7.67		.31	
	AMC	10.69		5.58 to 15.79		<.001		13.82			8.19 to 19.44		<.001	
	Surgeon 1	–2.87		–7.60 to 1.86		.23		–10.36			–15.36 to –5.36		<.001	

^a^ACT: anesthesia-controlled time.

^b^SCT: surgery-controlled time.

^c^ASA: American Society of Anesthesiologists.

^d^ACT for total hip arthroplasty had 609 observations and an *R*^2^/*R*^2^ adjusted value of 0.061/0.056; and total knee arthroplasty had 624 observations and an *R*^2^/*R*^2^ adjusted value of 0.037/0.003.

^e^SCT for total hip arthroplasty had 609 observations and an *R*^2^/*R*^2^ adjusted value of 0.043/0.039; and total knee arthroplasty had 624 observations and an *R*^2^/*R*^2^ adjusted value of 0.060/0.055.

^f^AMC: academic medical center.

**Table 5:** Multivariable linear regression coefficients for the association of ACT^a^ and SCT^b^ with anesthesia, hospital, and surgeon.

Outcome and variable		Total hip arthroplasty^c^				Total knee arthroplasty^d^		
		Estimates (min)	95% CI	*P* value	Estimates (min)		95% CI	*P* value
**ACT**								
	Coefficient intercept	22.15	19.29 to 25.01	<.001	19.35		16.91 to 21.78	<.001
	Neuraxial anesthesia	1.38	–0.71 to 3.47	.92	1.75		–0.21 to 3.70	.11
	AMC^e^	4.08	2.08 to 6.07	<.001	3.83		2.21 to 5.64	<.001
	Surgeon 1	–2.12	–3.92 to –0.32	.02	–1.56		–3.05 to –0.07	.04
**SCT**								
	Coefficient intercept	105.56	98.01 to 113.11	<.001	103.01		94.93 to 111.20	<.001
	Neuraxial anesthesia	–1.18	–6.71 to 4.35	.68	–0.53		–7.09 to 6.03	.87
	AMC	10.88	5.62 to 16.15	<.001	13.93		8.17 to 19.70	<.001
	Surgeon 1	–3.17	–7.93 to 1.59	.19	–10.34		–15.34 to –5.35	<.001

^a^ACT: anesthesia-controlled time.

^b^SCT: surgery-controlled time.

^c^ACT for total hip arthroplasty had 609 observations and an *R*^2^/*R*^2^ adjusted value of 0.036/0.031; and total knee arthroplasty had 625 observations and an *R*^2^/*R*^2^ adjusted value of 0.037/0.033.

^d^SCT for total hip arthroplasty had 609 observations and an *R*^2^/*R*^2^ adjusted value of 0.033/0.028; and total knee arthroplasty had 625 observations and an *R*^2^/*R*^2^ adjusted value of 0.058/0.054.

^e^AMC: academic medical center.

## Discussion

### Overview

A paucity of literature exists for benchmarking operative times in different surgical settings, and our study therefore aimed to refine the prediction of surgical case duration for THA and TKA between an academic center and a CH for the same orthopedic surgeons. Our results showed that both SCT and ACT were statistically significantly longer for primary hip and knee arthroplasty at the AMC compared with the CH. The mean ACT was higher at the AMC by less than 4 minutes for THA and TKA for both surgeons, and this modest increase in ACT when trainees are present is consistent with previous reports [12,13]. Therefore, although the participation of anesthesia trainees at the AMC may elongate the ACT, these results are not clinically meaningful in the context of OR efficiency—decreases in ACT have not been shown to permit the scheduling of another OR case in a workday but may be relevant for patient satisfaction and experience [14]. In addition, it is crucial to recognize the value of surgical training and its pivotal role in preparing the next generation of health care providers. Finding a balance between providing trainees with comprehensive experiences while maintaining operational efficiency is crucial.

The mean SCT was greater at the academic center for THA and TKA procedures compared with the CH. Our results may have clinically significant implications, as a 16-minute difference in 4 cases can result in an extra hour of operating time per day, allowing for the scheduling of another short case during a normal surgical block or relieving staff in the OR earlier to reduce overtime call coverage pay. Previous studies have shown that operative time significantly increases when procedures are performed with surgical resident or surgical fellow participation [15,16]. The *R*^2^ values in our results (<10%, Tables 3-5) also indicate the existence of other covariates that were not adjusted for in our multiple linear regression modeling such as the presence of scrub technician trainees, anesthesia trainees, surgical trainees, or traveling nursing staff who are not regularly participating in orthopedic surgery cases at the hospital. Therefore, understanding this cost of training surgical residents, nursing, and scrub technician staff can help OR managers find a balance between achieving scheduling and financial targets while exploring strategies to provide adequate educational opportunities.

Furthermore, it is pragmatic to identify other factors that could affect OR efficiency (ie, type of anesthesia, performing secondary procedures during the joint replacement, or performing bilateral procedures). In this study, we observed no significant differences between the ACT or SCT for both surgical centers when comparing general anesthesia versus neuraxial anesthesia. The current literature offers mixed results about the effect of anesthesia type on surgical time. A meta-analysis comparing the use of neuraxial anesthesia versus general anesthesia found no significant differences in surgical time for a variety of cases [17]. Contrastingly, a different study found that spinal anesthesia significantly reduced the duration of TKA surgery and resulted in decreases in the rates of thromboembolic events, infections, blood transfusion rates, and hospital length of stay [18]. Another study also found significant decreases in ACT when regional anesthesia was used [19-21]. Furthermore, there is limited literature exploring the implication of ASA classification on SCT or ACT. Previous studies propose a positive correlation between ASA classification and perioperative complication rates for patients undergoing fixation of hip fractures [22]. ASA classification is also a significant predictor of length of stay cost for patients undergoing TKA [23,24]. In our study, there was an increase in ACT and SCT by approximately 4 and 6 minutes respectively for both surgical centers when the patient had moderate to severe systemic disease (ASA class III or IV) compared with patients with mild or no systemic disease (ASA class I or II). With over 3700 primary joint arthroplasty cases performed across the AMC’s hospitals per year, a 10-minute decrease in ACT and SCT per case could result in 37,000 available OR minutes, equating to greater than 200 additional orthopedic cases (at an average of 155 minutes per case).

### Limitations

Our study has several limitations. One of the limitations of this study is the sample size. Even with 1234 cases, there was still an underrepresentation of patients with ASA classifications of I and IV. While we feel this sample represents the patient population that normally receives primary joint replacement surgery, a larger cohort would allow for a more granular analysis of each ASA classification group. A second limitation is associated with the generalizability of this study. Our analysis was performed at 1 AMC and 1 CH. Only 2 surgeons were tracked for this study due to their unique movement between the 2 clinical sites. A larger cohort of surgeons with a similar multisite practice pattern could provide data that would be more generalizable. Furthermore, the perioperative environment and considerations at other academic and CHs could lead to different results. Therefore, the increased difference seen in SCT in our study could be a result of differences in OR culture between academic institutions and CHs, along with increased time required for on-the-job education for trainees in nursing and scrub technicians. Individual variation in the documentation of the surgery process could also be a confounding variable for the calculation of ACT and SCT. In addition, the decision-making process regarding the choice of surgical center involves a complex interplay of patient and surgical factors, some of which may not have been captured in our analysis. For example, the selection of cases for the academic center hospital may be influenced by factors such as case complexity, patient comorbidities, or surgeon preference. These potential biases could introduce uncontrolled variability into the ACT or SCT. Last, we define *Ready for Positioning* as the time point when anesthesia had completed its activities and when the patient was prepared for surgical positioning including completion of any additional intravenous lines or invasive monitoring if required for the procedure. However, other logistical factors may influence the actual commencement of surgery. Therefore, although our definition represents the point when anesthesia activities were complete, it does not imply the presence and readiness of the surgical team. Future directions of this study include assessing the effect of different levels of trainee and surgical nursing team involvement in our analysis, in addition to comparisons of cost and clinical outcomes between the 2 hospital locations and postoperative outcomes including complication rates.

### Conclusions

OA is 1 of the 10 leading causes of disability in developed countries and the consequential growth in the volume of hip and knee replacement surgeries to manage end-stage OA will contribute to substantial and rising health expenditure [25,26]. Therefore, it is critical to optimize OR scheduling and management to maximize efficiency and decrease costs for both health systems and patients. As the demand for THA and TKA grows, it will be increasingly important to optimize OR efficiency for those surgeries. This study aims to validate and compare benchmarking times for ACT and SCT in a single patient population in both an academic center and a CH. One major application of these findings is that there is an efficiency benefit of performing primary joint replacements in our CH, as demonstrated by an average 16-minute reduction of SCT and a 4-minute reduction of ACT per case. This equates to a savings of approximately 80 minutes over the course of 4 surgical cases in a day, which could allow for the scheduling of another case. Such data can help to increase the reliability of surgical duration predictions and optimize scheduling to ultimately improve OR use, reduce cost, and improve patient experience.

## Appendix

Multimedia Appendix 1 STROBE (Strengthening the Reporting of Observational Studies in Epidemiology) checklist.

## Abbreviations

AA: anesthesiology assistant
ACT: anesthesia-controlled time
AMC: academic medical center
ASA: American Society of Anesthesiologists
CH: community hospital
CPT: current procedural terminology
OA: osteoarthritis
OR: operating room
SCT: surgery-controlled time
STROBE: Strengthening the Reporting of Observational Studies in Epidemiology
THA: total hip arthroplasty
TKA: total knee arthroplasty

## Data Availability

The data sets generated or analyzed during this study are available from the corresponding author on reasonable request.
